# Moses Gunn, A.M., M.D.

**Published:** 1867-02

**Authors:** 


Moses Gunn, A.M., M.D., lately elected to the Chair of Principles and Practice of Surgery and Clinical Surgery, vacated by the death of Prof. Brainard, scarcely needs any introduction to the medical profession of the North-west. From the organization of the Medical Department of the University of Michigan, he has filled the same chair in that institution, and its success and popularity have been largely due to the ability with which he has discharged its duties.

Prof. Gunn gave the first systematical and complete course of anatomy ever given in Michigan, some three years or more before the organization of the Medical Department of the University. His lectures upon the cadaver were attended by a class of some thirty medical students, and also by a large number of scientific and professional gentlemen. These lectures secured him the position in the University which he has continued to hold from 1850 down to the present session of the Medical Department, where he is at present lecturing to a class of over five hundred students.

Notwithstanding this large attendance, and whilst still at the zenith of his popularity in the institution, he has recognized in full the wider field for usefulness afforded in Chicago, in accepting the appointment in Rush Medical College, and will remove to this city early in the ensuing spring, to enter upon the active duties connected with his new position, and the surgical requirements of the North-west.

The writer of the present notice, who has known him long and intimately, ventures, over his initial, to write thus:—

Prof. Gunn's success, as a surgeon and teacher, has been secured by his exceedingly minute and accurate acquaintance with anatomy, combined with exquisite sagacity in diagnosis; a cool head; steady muscle; and great mechanical genius. He is never at a loss for apparatus, and invents models which would make the fortune of a patent right dealer, off hand. His straps and splints, springs and bandages, *et id omne genus*, always fit the variety of the species, and not merely the class or order. He has peculiar skill in the use of the knife, but rather secures success against the chances of failure by careful attention to those indispensable details which, too often, being neglected cause "brilliant operations" to be succeeded by ignominious results.

Dr. G. was, for about a year, connected with the Army of the Potomac, when disease was most virulent and wounds the most frequent, in the bloodiest and deadliest days of the ill-starred first peninsular campaign. But to such an extent did his arduous labors exhaust his system, that he was obliged to return to his post as a teacher. His physical health has scarcely yet recovered from the shock then received, but the widened experience thus gained in *his* possession becomes invaluable.

Upon the basis of a thorough general education, he has built up an enviable structure of professional knowledge. Fluent in speech, whether in the vernacular or the German, he is equally at home in private conversation or in the crowded lecture-room. With somewhat rapid enunciation and great energy of style, he combines a methodical and exhaustive arrangement of the details of his subject, which is developed with impressive clearness and exactness of language. Personal and professional popularity has thus far attended him, and, the writer believes, will be added to by the present change in his relations. Still in the prime of life, with a commanding and powerful *physique*, untiring energy and earnest devotion to his profession; with Chicago and the great North-west for his field, and Rush Medical College for his forum, he has a career worthy of his efforts before him.

